# Leczyme: A New Candidate Drug for Cancer Therapy

**DOI:** 10.1155/2014/421415

**Published:** 2014-04-23

**Authors:** Takeo Tatsuta, Shigeki Sugawara, Kohta Takahashi, Yukiko Ogawa, Masahiro Hosono, Kazuo Nitta

**Affiliations:** ^1^Division of Cell Recognition Study, Institute of Molecular Biomembrane and Glycobiology, Tohoku Pharmaceutical University, 4-4-1 Komatsushima, Aoba-ku, Sendai 981-8558, Japan; ^2^Divisions of Microbiology and Functional Morphology, Department of Pharmacy, Faculty of Pharmaceutical Science, Nagasaki International University, 2825-7 Huis Ten Bosch, Sasebo, Nagasaki 859-3298, Japan

## Abstract

Sialic acid-binding lectin (SBL), isolated from oocytes of *Rana catesbeiana*, is leczyme and has both lectin and ribonuclease (RNase) activities. A remarkable antitumor effect of SBL has also been reported. SBL agglutinates various kinds of tumor cells but not normal cells. SBL agglutination activity is not affected by mono- or oligosaccharides. However, SBL-induced agglutination and antitumor effects are inhibited by sialomucin but not asialomucin. In addition, SBL has very little effect on sialidase-treated cells. SBL causes cancer-selective induction of apoptosis by multiple signaling pathways, which target RNA. Synergistic antitumor effects with other molecules, such as tumor necrosis factor-related apoptosis ligand (TRAIL) and interferon-**γ** (IFN-**γ**), have been reported. Thus, SBL may be a novel candidate molecule for anticancer drug development. Sialoglycoconjugates on the tumor cell surface may be associated with lectin activity and antitumor effects of SBL. We review the properties of SBL, particularly its lectin, RNase, and antitumor activities, and comprehensively examine the potential application of SBL for clinical purposes.

## 1. Introduction

The recent progression of protein engineering and bioinformatics technology has enabled scientists to explore functional genes from genome sequences, to construct engineered proteins from artificially constructed DNA, and to analyze protein functions both* in vitro* and* in vivo*. Furthermore, it has been revealed that several biomolecules such as proteins and lipids are glycosylated, and glycosylation may regulate the function of biomolecules. Glycosylation plays an important role in individual survival because knocking out some glycogens is lethal [1]. Therefore, it is important to analyze the glycosylation properties of biomolecules to more fully understand their functional properties. The concept of glycomics, that is to say, the elucidation of involvement of the carbohydrate chains in various biological phenomena such as cell-cell recognition, adhesion, differentiation, proliferation, and senescence, has begun to be indicated in the 1960s. Although the increment of sialic acid in serum of cancer or inflammatory disease patients was reported from the 1950s, the fact that abnormal glycosylation patterns are observed in malignant transformation of cells was discovered in the late 1960s [2]. Glycosylation changes associated with oncogenic transformation were first demonstrated with glycolipids and subsequently glycoproteins. In the 1980s, preparations of monoclonal antibodies that react with cancer cells, but were less reactive with normal cells, were developed. Most of these antibodies reacted with glycan; therefore, glycoantigens began to attract attention as tumor-specific markers. In the 1990s, it was revealed that glycan not only was a marker but also played a key role in cancer migration, metastasis, and invasion [3]. At present, antibodies and proteins that react with cancer-associated glycoantigens are utilized for diagnosis or prognostication and are expected to be further developed for therapeutic applications.

Lectins are among the molecules that recognize glycoconjugates. Lectins exist universally in plants, microorganisms, and animals, and their functions significantly contribute to our understanding of glycoinformation in cellular biology. The first functional discovery of lectin, from seeds of* Ricinus communis*,was its ability to agglutinate red blood cells [4]. Major investigations of lectins from plants followed this discovery. Interest was primarily on the value of lectins as tools for detecting carbohydrate chains with a focus on its binding ability to specific carbohydrate structures. Nowell (1960) observed that* Phaseolus vulgaris* lectin (PHA-L) binds to glycoconjugates on lymphocyte cell surfaces and shows mitogenic activity [5]. Furthermore, Aub et al. (1963) reported that* Triticum vulgaris* lectin (wheat germ agglutinin, WGA) binds to glycoconjugates on cancer cell surfaces and displays agglutination activity [6]. These discoveries put lectin research in the spotlight. In 1980, Goldstein et al. (1980) defined lectin as “a sugar-binding protein of nonimmune origin that agglutinates cells or precipitates glycoconjugates” [7]. Ashwell and Harford (1982) subsequently observed a protein on the hepatocyte cell surface that binds to asialoprotein, which has galactose on the nonreduced terminus, and revealed that the protein is a carbohydrate-specific endocytosis receptor [8]. This finding initiated substantial research into animal lectins. It is now known that fecundation, development, immunity, and virus infection are attributed to interactions between carbohydrates and proteins such as lectin that specifically recognize the carbohydrate. Thus, lectins have captured a vast scientific audience through their ability to be a recipient for carbohydrates and to act as information molecules in living organisms. At present, in the postgenome era, lectins are utilized in leading-edge techniques such as glycome analysis [9, 10].

Historically, replicative DNA was the main target of anticancer agents. The origins of chemotherapeutic drugs are believed to be nitrogen mustards (alkylating agents) that were used as chemical weapons in World War II [11, 12]. Since then, several chemotherapeutics have been exploited on the basis of their ability to alter DNA replication. In the 20th century, these drugs can be categorized into alkylating agents, platinum-containing drugs, antimetabolites, topoisomerase inhibitors, or microtubule inhibitors. The development of these agents made a great contribution to the progress of cancer therapy. However, side effects (because of their low selectivity) and resistance to the drugs caused problems. In the 21st century, drugs with molecular targets have been developed. For example, imatinib targets the Philadelphia chromosome-positive (Ph+) leukemia [13], and the anti-human antibody preparation rituximab targets cluster of differentiation (CD) 20 that is only expressed in B cells [14]. Therefore, it is apparent that new agents are being developed to improve the selectivity of biological mechanism-based agents, to overcome tumor resistance, and to minimize the toxic effects on normal cells [15].

Ribonucleases (RNases) are enzymes that catalyze RNA degradation. RNases have various biological roles such as nutritional function, remobilization of phosphate, senescence, self-incompatibility, defensin-like activity, and RNA metabolism [16, 17]. Some RNases display angiogenic, neurotoxic, antitumor, or immunosuppressive activities [18]. Bovine pancreatic RNase A (EC 3.1.27.5) [19] was the first RNase shown to have anticancer activity* in vitro* and* in vivo* [20–25]. High amounts of RNase A were required to elicit anticancer activity; however, more effective RNases have been recently reported [26]. The proposed mechanisms of RNase-induced cytotoxicity are (i) cell surface binding and internalization, (ii) translocation to the cytosol, (iii) evasion of the cytosolic RNase inhibitor protein (RI), and (iv) degradation of cellular RNA. Differences in the efficiency of any of these steps can affect cell susceptibility [27]. Onconase (ONC), an RNase isolated from* Rana pipiens* oocytes, is a promising RNase for cancer therapeutic drug development [28]. ONC manifests cytotoxic and cytostatic effects [29]. It also has synergistic actions with other agents such as tumor necrosis factor (TNF)-*α*, vincristine, and tamoxifen [30–34]. Furthermore, ONC had been utilized in clinical trials for the treatment of malignant mesothelioma and non-small-cell carcinoma [35]. ONC has beneficial properties for potential clinical applications and these include the following: (i) evading human RNase inhibitors in the cytosol, (ii) inhibitory activity against broad types of human tumors, (iii) no apparent influence on the immune response, and (iv) exertion of only weak and reversible renal toxicity [36]. In addition to the discovery of other RNases that exhibit antitumor effects, such as binase isolated from* Bacillus intermedius* [37], further investigations into anticancer RNases and genetic engineering of known RNases may reveal new RNase applications for anticancer therapeutics [17, 36, 38, 39].

Cell recognition properties of some lectins may be promising avenues to explore to develop anticancer therapeutics. Kawauchi et al. (1975) discovered a lectin from the oocytes of* Rana catesbeiana* [40] that shows unique abilities to agglutinate a large variety of cancer cells but not normal cells. Cell agglutination caused by lectin was inhibited by glycoproteins or glycolipids (gangliosides) containing sialic acids. Thus, it was named sialic acid-binding lectin (SBL) from* R. catesbeiana.* SBL cell agglutination activity is attributed to the binding of SBL to glycoconjugates containing sialic acid on the cell surface. This is because SBL-induced agglutination is strongly inhibited by glycoproteins such as mucin, but not asialomucin, and sialidase-treated cells reduce the cell agglutination induced by SBL [41]. In addition, SBL shows homology to members of the RNase A superfamily and has pyrimidine base-specific RNase activity [42–44]. Therefore, SBL is an interesting multifunctional molecule that has both lectin and RNase activities and is consequently called a leczyme (lectin + enzyme) [45]. It is also notable that SBL has curious antitumor effects. The antitumor effect of SBL has been reported* in vitro* and* in vivo* [44]. We recently reported that SBL has antitumor effects on various cancer cells, including multidrug resistant (MDR) cells, but not on normal cells. We also demonstrated that SBL has synergistic effects with the TNF-related apoptosis-inducing ligand (TRAIL) [46, 47].* R. catesbeiana* RNase (RC-RNase), purified from* R. catesbeiana* oocytes by Liao et al. in 1992, is identical to SBL [48, 49] and also has cancer-selective antitumor activity and synergistic effects with interferon-*γ* (IFN-*γ*) [50, 51]. Taken together, SBL could be an innovative candidate drug for cancer therapy. In this study, we summarize the properties of SBL and discuss the possibility of developing it as anticancer drug.

## 2. SBL Lectin Activity

SBL was observed to be an agglutinin of tumor cells because it could selectively agglutinate various types of tumor cells but not normal cells, and this agglutination was inhibited by glycoconjugates containing sialic acids. SBL lectin activity is well documented by Nitta et al. (1987, 1994) [41, 44]. In addition, we tested the reactivity of SBL to 20 human and animal cancer cells and 10 normal cells and observed that the majority of cancer cells showed high reactivity to SBL, while normal cells (fibroblast, lymphocytes, and erythrocytes) exhibited low reactivity (10%–50% reactivity compared to S-180 mouse ascites cells that were used as a standard) [41].

The cancer-specific reactivity of SBL facilitated research that unveiled the factors that affect the SBL agglutination activity. These factors include inhibition by saccharides, glycoproteins, glycosaminoglycans, and polyamines (Table 1). SBL agglutination activity is not affected by saccharides, except N-acetylneuraminyllactose (50 mM) [41]. In contrast, glycoproteins that contain sialic acids, such as mucin or fetuin, strongly inhibit SBL agglutination activity. These inhibitory effects are reduced when the glycoproteins are treated with sialidase. For the glycosaminoglycans tested, heparin shows strong inhibitory effects. It seems that heparin has an effect on SBL-induced agglutination as a substrate analog and binds to the cell binding site of SBL. There is also the possibility that negative charge components affect SBL activity, but chondroitin sulfate, keratin sulfate, or hyaluronic acids have less or no inhibitory activity. Thus, it is believed that the particular structure recognized by SBL may reside in the molecules. Polyamines inhibit the cell agglutination, and it was proposed that they interact with negatively charged cell surface components, including sialosyl residues (i.e., lectin-binding region), that are influenced by treatment with polyamines. Along with the reduction in inhibitory activity of SBL-induced agglutination by the sialidase-treated glycoproteins (asialoglycoproteins), sialidase treatment of cells reduces SBL agglutination activity. The sialidase-dependent reduction in tumor cell agglutination is inhibited by the copresence of sialidase inhibitors [44]. These results suggest that tumor cell agglutination occurs because of the recognition of sialic acid-containing molecules on cell surface by SBL.

Thus far, SBL does not show a sequence homologous to any other lectin. There are still several uncertainties regarding SBL lectin activity, particularly with regard to target structures and the binding mechanism. However, Irie et al. (1998) reported two putative sialic acid-binding sites of SBL at the N-terminus and a loop consisting of amino acid residues 57–75 [52]. This discovery may be the key to revealing the binding mechanism of SBL.

## 3. RNase Activity of SBL

**Figure 1:**
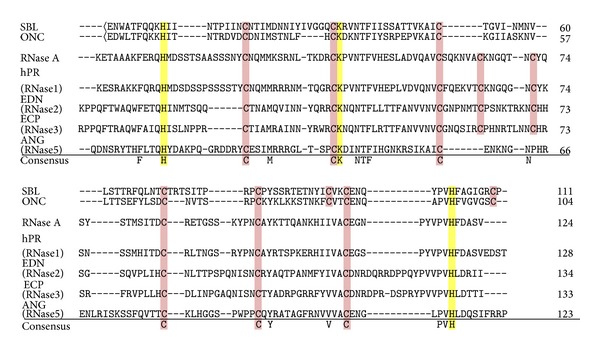
Amino acid sequence alignment of various members of the RNase A superfamily consensus amino acids is indicated at the bottom of the matrix. Amino acid residues that are essential for catalysis are denoted in yellow boxes and half-cystine residues are noted in pink boxes. <E indicates pyroglutamic acid. Figures to the right of the matrix are the amino acid numberings. hPR, human pancreatic RNase; EDN, eosinophil-derived neurotoxin; ECP, eosinophil cationic protein; ANG, angiogenin.

The amino acid sequence of SBL consists of 111 residues [53], and we reported that SBL exhibits high homology with angiogenin and pancreatic RNases [42]. It is now confirmed that SBL has homology with various members of the RNase A superfamily (Table 2). RNases can be categorized into RNase A, RNase T1, RNase T2, RNase H, RNase L, and RNase P families, among others [16, 36]. Members of the RNase A superfamily proteins are typically composed of approximately 130 amino acids. They show RNA-cleavage activity for pyrimidine, at an optimum pH of 7-8. They have three catalytic residues (a lysine and two histidines) and 6–8 cysteines that form three to four disulfide bonds [54]. Sequence identities vary from 20% to almost 100%, and their three-dimensional structures closely resemble each other. The amino acid sequence alignment is shown in Figure 1.

SBL has conserved catalytic amino acids residues at the same positions as other RNase members. It also has eight cysteines that form four disulfide bonds. The actual RNase activity of SBL is essentially a pyrimidine base-specific RNase and detailed studies of substrate specificities have been performed [43]. While cow (RNase A), turtle, and chicken RNases are essentially cytosine base-preferential [55, 56], SBL appears to be a uracil-preferential RNase because hydrolysis rates of the dinucleotide phosphate UpX consistently exceed that of CpX (X = A, G, U, and C) [57]. This is also similar to ONC and iguana RNase [57]. When comparing RNase activity of ONC on yeast RNA with RNase A in sodium acetate buffer (50 mM, pH 5.5), ONC shows approximately 10% activity of RNase A [58]. We support this finding because we observed that SBL has approximately 40% RNase A activity (T. Tatsuta, M. Hosono, S. Sugawara, unpublished observation). Although RNase activities of SBL and ONC are relatively weaker than RNase A, their binding affinities to the human RNase inhibitor (RI) are extremely low compared with RNase A. These properties may contribute to the antitumor effects of SBL. In contrast, RNase A, which shows high affinity to human RI, does not appear to elicit antitumor activity.

## 4. Antitumor Activity of SBL

SBL antitumor activity has been reported* in vivo* and* in vitro* [44]. SBL inhibits solid tumor growth and ascites accumulation in mice inoculated with Ehrlich cells and sarcoma 180 cells [44]. SBL also prolongs the life span of tumor-bearing mice [44]. SBL exhibits cytotoxicity against mouse leukemia P388 and L1210 cells* in vitro*, although RNase A does not show toxicities either* in vitro* or* in vivo* [44].It is believed that the antitumor activity of SBL arises from the coordination of lectin activity that recognizes glycoconjugates containing sialic acids on tumor cell surfaces and RNase activity that decomposes RNA required for cell survival [59].

The addition of benzyl-*α*-N-acetylgalactosamine and SBL to a culture medium leads to resistance of cells to SBL. This suggests that the internalization of SBL may be mediated by O-linked carbohydrate chain(s) of the glycoconjugates [52]. We established the SBL-resistant P388 cell variant RC-150 [60]. The doubling time, tumorigenicity, and lethality of RC-150 cells were similar to those of P388 cells. SBL agglutinated both P388 and RC-150 cells, and no difference was observed between the sialidase-labile sialic acid levels in RC-150 and P388 cells. Although SBL had no effect on growth of RC-150 cells, even at a concentration of 100 *μ*M, the 50% inhibitory concentration for growth of P388 cells was approximately 3.1–6.2 *μ*M. A study utilizing dansylcadaverine- (DC-) labeled SBL indicates that RC-150 cells have a defective mechanism of internalization [60]. This finding underscores the importance of not only binding to the cell surface but also the internalization into cells to exert the antitumor effects of SBL [60].

We recently reported the validity of SBL for human leukemia cell lines and the detailed mechanism of SBL-induced cell death [46]. SBL manifests cytotoxicity in several kinds of human leukemia cell lines, including MDR cells [46]. However, conventional DNA-damaging clinical agents, such as etoposide (ETO) and doxorubicin (DOX), do not show cytotoxicity to MDR cells. SBL induces apoptosis. For example, SBL-treated cells present typical apoptotic morphological alterations, including karyorrhexis, nuclear condensation and fragmentation, and apoptotic biological changes such as phosphatidylserine (PS) externalization, activation of caspases, and DNA fragmentation. This SBL-induced DNA fragmentation is completely blocked by a pan-caspase inhibitor, carbobenzoxy-valyl-alanyl-aspartyl-[O-methyl]-fluoromethylketone (z-VAD) [46]. This suggests that the cytotoxicity of SBL is induced in a caspase-dependent manner. To identify the SBL-induced signaling pathway, we performed studies using a combination of specific caspase inhibitors and mitochondrial membrane depolarization-detecting reagents. We clearly showed that SBL-induced mitochondrial depolarization is not diminished by z-VAD, while TRAIL-induced mitochondrial depolarization is completely inhibited by z-VAD [46]. This indicates that cytotoxicity of SBL is induced through caspase-dependent apoptosis whereby the mitochondrial perturbation occurs as an upstream event. The implication for endoplasmic reticulum (ER) stress, a new target for cancer therapy, is also apparent [61]. SBL induces an unfolded protein response (UPR) [61]. In addition, the involvement of ER stress-mediated apoptosis is indicated because inhibition of caspase-4, the initiator caspase in ER-stress mediated apoptosis, diminishes SBL-induced apoptosis [61]. A further experiment using specific caspase inhibitors indicates that caspase-9 activation is partially involved in caspase-4 activation but that mitochondria perturbation and ER stress occur independently in SBL-treated cells. This is because SBL-induced mitochondrial membrane depolarization or UPR is not affected by caspase-9 or caspase-4 inhibition, respectively. These results suggest that SBL causes apoptosis by multiple apoptotic pathways that involve ER stress and mitochondrial pathways. Furthermore, a comparative study with thapsigargin (TG), a strong ER stress inducer and apoptosis mediator, indicates that a mitochondrial pathway may be intensely involved in apoptosis induced by SBL [61].

SBL has synergistic antitumor effects with other molecules. For example, IFN-*γ* synergizes SBL-induced cell death against leukemia, breast carcinoma, and in hepatoma cell lines [50, 51]. In our recent report, we assessed the efficiency of SBL for treatment of malignant mesothelioma and its synergistic effects with TRAIL [47]. We showed that SBL inhibits cell growth of various malignant mesothelioma cells, but not of nonmalignant mesothelial cells. The combinatorial treatment with SBL and TRAIL induced synergistic apoptosis in malignant mesothelioma cells. Further studies revealed that the cotreatment of SBL and TRAIL enhances the cleavage of Bid, a proapoptotic member of the Bcl-2 family, that is cleaved by activated caspase-8 and subsequently causes an efflux of cytochrome c from the mitochondria. Thus, it is suggested that apoptotic signaling is amplified by an amplification loop consisting of caspase activation, mitochondria perturbation, and truncation of Bid. This synergistic, cytotoxic effect of SBL and TRAIL is also not observed in nonmalignant mesothelial cells. Therefore, it is suggested that the combination of SBL and TRAIL could be an effective treatment for malignant mesothelioma.

**Figure 2:**
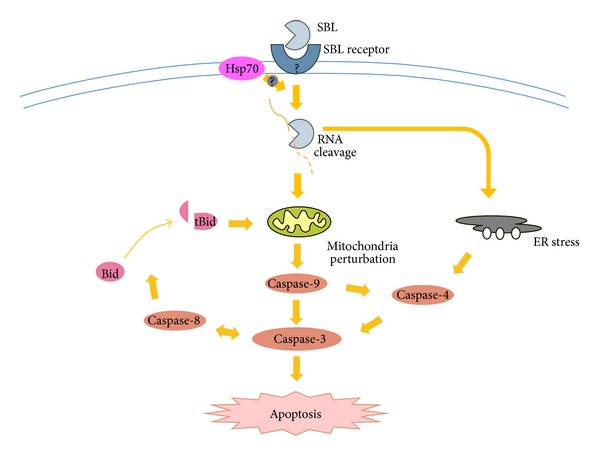
Proposed model for apoptotic mechanisms induced by SBL. SBL induces cancer-selective apoptosis through the SBL receptor by multiple signaling pathways in which RNA is its target.

To summarize, the proposed apoptotic signal transduced by SBL is shown in Figure 2. In brief, SBL binds to the tumor cell surface and internalizes into the cells. SBL degrades cellular RNA and triggers mitochondrial perturbation and also ER stress. Subsequently, an apoptotic signal is amplified by caspase activation that leads to cell death. Bid cleavage contributes to the synergistic apoptosis-inducing effect of TRAIL. This mechanism suggests that SBL has an innovative antitumor effect that can cause cancer-selective induction of apoptosis by multiple signaling pathways, in which RNA is its target.

## 5. Target(s) of SBL on the Cell Surface

The antitumor effect of SBL is inhibited by mucin but not asialomucin [41]. Furthermore, treatment of cells with sialidase also diminishes the antitumor and lectin activities of SBL [43]. This suggests that SBL binds to glycoconjugates containing sialic acids on the cell membrane that are responsible for the apoptosis-inducing effect of SBL [48, 62]. It has recently been demonstrated that methyl-*β*-D-cyclodextrin (M*β*CD) inhibits the effect of SBL on the viability of P388 cells [62]. M*β*CD disrupts the construction of cholesterol-rich microdomains on the cell surface that contains several types of glycosphingolipids (GSLs) and glycoproteins. Thus, the target molecule of SBL is most likely located in cholesterol-rich microdomains on the P388 cell surface [62].

Heat shock proteins (Hsp) have also been implicated in SBL-induced cytotoxic effects [62, 63]. Hsp 70 and Hsc 70 are Hsp 70 family proteins, and it has been revealed that they are involved not only in heat shock responses but also in cell death [15, 64–66]. They are located in the cytosol and migrate to the nucleus after specific stress [67]. However, they are also expressed on the cell surface and interact with various types of receptors [68–70]. In addition, Hsps, such as Hsp 70 and Hsc 70, are expressed in the glycosphingolipid-enriched microdomain (GEM) on the cell surface [71, 72]. Hsc 70 and integrin *α*v*β*3 form a complex in the GEM and act as a receptor for rotavirus and may participate in the process of adsorption and penetration of viruses into cells [73]. Although SBL is able to bind to Hsp 70, which is expressed in GEM on P388 cell surface, there are no reports showing sialylated Hsp 70. The binding of SBL to P388 cell membrane is not affected by a decrease in Hsp 70 expression using quercetin, but an attenuated induction of apoptosis is apparent. These results suggest the possibility that Hsps on the P388 cell surface are not the receptors of SBL. Instead, Hsps may interact with the SBL receptor or participate in the penetration of SBL into cells. This may affect the cytotoxicity of SBL because cell susceptibility to RNase can be affected by binding as well as the internalization or translocation of RNases as described above.

## 6. Potential Medicinal Application of Lectins and RNases

Research into glycobiology is rapidly progressing. Lectins are potent biomolecules that could be used as tools to explore novel tumor markers or diagnostic agents primarily because of their ability to recognize specific carbohydrates. Narimatsu et al. (2010) constructed a system for the verification of candidate molecules that exhibit disease-specific glycoalterations. This was achieved by applying glycoproteomics-assisted strategies that include lectin chromatography, lectin staining, and lectin microarray. In doing so, they discovered a useful cholangiocarcinoma marker [74, 75]. Akatsuka et al. (2010) proposed a diagnosis platform using antibodies and engineered lectins for detecting increases in glycoprotein expression and alterations of sugar moieties that accompany with tumor progression [76–79]. Moreover, some plant lectins, such as concanavalin A (jackbean lectin) and mistletoe lectin, induce autophagy of cancer cells and thus have been drawing increasing attention with regard to their antitumor activity and potential clinical application in cancer therapeutics [80–82].

On the cell membrane, sialic acids are generally observed to be linked to the terminal position of the carbohydrate groups of glycoproteins and glycolipids and play important roles in various biological processes such as conformation, recognition, or binding of glycomolecules [83]. Changes in glycosylations are known features of cancer cells. It has been suggested that altered sialylation is closely associated with malignant phenotypes, including metastasis and invasiveness [84, 85]. In addition, there are several reports that propose the involvement of sialic acids in cancer. For example, a high level of sialic acid is associated with the metastatic potential of gastric cancer cells [86], and inhibition of cancer cell growth and motility is analyzed using a lectin from the seeds of* Maackia amurensis* (MASL) that has affinity for sialic acid [87]. Some mechanisms remain to be elucidated with regard to the lectin activity of SBL, but the implication of binding activity against sialic acid-containing glycoconjugates is likely from the study on agglutination inhibition. This mechanism may be involved in the antitumor effects of SBL and may be cancer-selective.

Because the antitumor effects are attributed to a new mechanism, cytotoxic RNases are attracting attention as potent anticancer reagents or alternatives to conventional DNA-damaging anticancer drugs. Investigations to find novel medicinal RNases, regardless of the species, have been actively performed and have identified several new RNases such as RNase from* Lyophyllum shimeji* [88], RNase MC2 from* Momordica charantia *[89], and Amphinase from* R. pipiens* [90] that exhibit antitumor effects. On the contrary, efforts to construct the effective variants or RNases-fusion molecules to make RNase more selective or stronger are now taking advantage of the latest genetic and protein engineering techniques. Investigators are constructing RNases that link to antibodies, such as anti-CD74 and anti-human epidermal growth factor receptor-related 2 (HER2) [91, 92], to examine selective anticancer effects. Others are attempting to induce human RNases to manifest antitumor activity by fusion with a nuclear localization signal that enables human RNases to evade an RNase inhibitor in cytosol and exerts its RNase activity in nucleus [93]. Finally, scientists are also trying to reduce organ-specific toxicity of particular RNases by fusion with albumin because albumin prevents the accumulation of RNase in particular organs [94]. All these innovative investigations indicate the potential effectiveness of RNases for cancer therapy. Nevertheless, there are still many mechanisms to be elucidated, particularly with regard to cancer selectivity and the involvement of signal transduction pathways after RNA degradation.

## 7. Conclusions

SBL is a leczyme that has both lectin and RNase activities. SBL has remarkable antitumor effects that are generating attention and leading to the development of new strategies to identify target molecules such as cellular RNAs. The antitumor effects of SBL are cancer-selective and are induced through multiple apoptotic signaling pathways that are implicated by mitochondria perturbation and ER stress. SBL also exerts synergistic antitumor effects with other anticancer molecules. These studies provide valuable insights and indicate the potential of SBL as a new type of anticancer drugs. Further study to explore the mechanisms of the SBL-induced antitumor effects, particularly the interaction of SBL with its target glycoconjugate, may open new avenues for the development of innovative clinical applications.

## Figures and Tables

**Table:** (a)

Mono- and oligosaccharides	Inhibitory effect
Xylose	−
D-Arabinose	−
L-Rhamnose	−
L-Fucose	−
D-Glucose	−
D-Galactose	−
D-Mannose	−
D-N-Acetylglucosamine	−
D-N-Acetylgalactosamine	−
Sucrose	−
Trehalose	−
Cellobiose	−
Melibiose	−
Lactose	−
Raffinose	−
N-Acetylneuraminyllactose	+

**Table:** (b)

	Concentration inhibiting 100% of lectin activity
Glycoproteins	(*μ*g/200 *μ*L)

Mucin	3.2–6.4^a^ (30^b^)
Asialomucin	70–140^b^
Fetuin	125–250
Asialofetuin	600–1000
Transferrin	>250
Asialotransferrin	>250
Ovomucoid	>250

Glycosaminoglycans	(*μ*g/200 *μ*L)

Heparin	0.25–0.5
Chondroitin sulfate A	>300
Chondroitin sulfate B	17–35
Chondroitin sulfate C	>300
Keratan sulfate	250
Hyaluronic acid	>300

Polyamines	(mM)

Putrescine	25–50
Spermidine	12.5
Spermine	3.1

Data were summarized from [40, 42]. All evaluation of inhibiting effect was done against SBL (25 ng/200 *μ*L)-induced agglutination of AH109A cells. In mono- and oligosaccharides, inhibitory effect was assessed negative (−) or positive (+). All saccharides tested here do not inhibit the lectin activity of SBL even at 100 mM, except N-acetylneuraminyllactose, which shows weak inhibition effect at 50 mM. In other compounds, the concentration that inhibits 100% of the lectin activity was indicated [(*μ*g/200) for glycoproteins and glycosaminoglycans and (mM) for polyamines]. Note that inhibitory effects of mucin and fetuin are reduced by sialidase treatment of them. ^a^Bovine submaxillary mucin (type I) from Sigma. ^b^Bovine submaxillary mucin from Worthington Biochemical Co.

**Table 2:** Protein sequence identity between SBL and various RNase A superfamily members.

Source	Frog		Bovine	Human			
Name	SBL	ONC	RNase A	hPR (RNase1)	EDN (RNase2)	ECP (RNase3)	ANG (RNase5)

Identity	—	49%	28%	26%	25%	25%	35%

hPR: human pancreatic RNase, EDN: eosinophil-derived neurotoxin, ECP: eosinophil cationic protein, ANG: angiogenin.
